# Roles of retrovirus-derived *PEG10* and *PEG11/RTL1* in mammalian development and evolution and their involvement in human disease

**DOI:** 10.3389/fcell.2023.1273638

**Published:** 2023-09-29

**Authors:** Hirosuke Shiura, Moe Kitazawa, Fumitoshi Ishino, Tomoko Kaneko-Ishino

**Affiliations:** ^1^ Faculty of Life and Environmental Sciences, University of Yamanashi, Yamanashi, Japan; ^2^ School of BioSciences, Faculty of Science, The University of Melbourne, Melbourne, VIC, Australia; ^3^ Institute of Research, Tokyo Medical and Dental University (TMDU), Tokyo, Japan; ^4^ Faculty of Nursing, School of Medicine, Tokai University, Isehara, Kanagawa, Japan

**Keywords:** PEG10, PEG11/RTL1, genomic imprinting, placenta, brain, innate immunity, human disease, mammalian development and evolution

## Abstract

*PEG10* and *PEG11/RTL1* are paternally expressed, imprinted genes that play essential roles in the current eutherian developmental system and are therefore associated with developmental abnormalities caused by aberrant genomic imprinting. They are also presumed to be retrovirus-derived genes with homology to the sushi-ichi retrotransposon GAG and POL, further expanding our comprehension of mammalian evolution via the domestication (exaptation) of retrovirus-derived acquired genes. In this manuscript, we review the importance of *PEG10* and *PEG11/RTL1* in genomic imprinting research via their functional roles in development and human disease, including neurodevelopmental disorders of genomic imprinting, Angelman, Kagami-Ogata and Temple syndromes, and the impact of newly inserted DNA on the emergence of newly imprinted regions. We also discuss their possible roles as ancestors of other retrovirus-derived RTL/SIRH genes that likewise play important roles in the current mammalian developmental system, such as in the placenta, brain and innate immune system.

## 1 Introduction

*Paternally expressed 10* (*PEG10*) and *PEG11/Retrotransposon Gag-like 1* (*RTL1*) were identified as paternally expressed genes in 2001 (Charlier et al., 2001; Ono et al., 2001) and demonstrated to play essential roles in the formation and maintenance of the placenta, respectively (Ono et al., 2006; Sekita et al., 2008). In addition to their biological importance for understanding the role of genomic imprinting in eutherians, *PEG10* and *PEG11/RTL1* have another critically important aspect: they are presumably derived from a retrovirus in the course of mammalian evolution. They share homology with the sushi-ichi retrotransposon *GAG* and *POL*, and were therefore termed RTL and/or sushi-ichi retrotransposon homolog (SIRH) after a long-terminal-repeat (LTR) retrotransposon. However, it is reasonable to assume that they are in fact derived from an extinct retrovirus that possessed a high degree of homology to the sushi-ichi retrotransposon. This is due to the fact that *PEG10* emerged in a therian common ancestor while *PEG11/RTL1* is a eutherian-specific gene although it is possible that its insertion occurred in a therian common ancestor but gained no function so has degraded over time (Suzuki et al., 2007; Edwards et al., 2008; Kaneko-Ishino and Ishino, 2012; 2015; Imakawa et al., 2022), and also that the gypsy type of LTR retrotransposon to which the sushi-ichi retrotransposon belongs is an infectious retrovirus in *Drosophila melanogaster* (Kim et al., 1994; Song et al., 1994). As newly exapted (acquired) genes, the emergence of *PEG10* and *PEG11/RTL1* were crucial in the emergence of the placenta in the course of evolution (Suzuki et al., 2007; Edwards et al., 2008). These facts have had a huge impact on developmental and evolutionary biology, because retrotransposons, such as LINEs, SINEs and LTR retrotransposon (including endogenous retroviruses), had long been recognized as “junk” in the mammalian genome. In addition, the simultaneous emergence of the *PEG10*-differentially methylated region (DMR) with *PEG10* in therian common ancestor (Suzuki et al., 2007; Kaneko-Ishino and Ishino, 2010) provides strong evidence that genomic imprinting arose as a defense mechanism against the insertion of foreign DNA (Barlow, 1993; Kaneko-Ishino et al., 2003; Kaneko-Ishino and Ishino, 2010). This is further supported by the finding that the newly integrated DNA sequences became DMRs in the most canonical imprinted regions that are conserved in eutherian mammals (Suzuki et al., 2011; Renfree et al., 2013; Kaneko-Ishino and Ishino, 2015; Kaneko-Ishino and Ishino, 2019; Kaneko-Ishino and Ishino, 2022).

*PEG10* and *PEG11/RTL1* are also very good examples of exaptation (domestication) during mammalian evolution (Kaneko-Ishino and Ishino, 2012). These two genes, together with *syncytin* derived from retroviral *ENVs* (Mi et al., 2000), open up a new field of research on the retrovirus-derived acquired genes that play important roles in the current mammalian developmental systems (Kaneko-Ishino and Ishino, 2012; Kaneko-Ishino and Ishino, 2015). Among the 11 RTL/SIRH genes in eutherians, only *PEG10* and *PEG11/RTL1* are imprinted, while the other RTL/SIRH genes are not. However, most are located on the X chromosome, and therefore exhibit monoallelic expression like imprinted genes (Brandt et al., 2005; Youngson et al., 2005; Ono et al., 2006). As the RTL/SIRH genes are functional in the placenta (Naruse et al., 2014), brain (Irie et al., 2015) and innate immunity against bacteria, viruses and fungi as microglial genes (Irie et al., 2022; Ishino et al., 2023), they have profoundly contributed to the establishment of certain eutherian-specific features. Most RTL/SIRH genes encode only GAG-like proteins, so it is possible that either *PEG10* or *PEG11/RTL1,* or both, are ancestral for other RTL/SIRH genes.

In this manuscript, we review the history of the *PEG10* and *PEG11/RTL1* research on genomic imprinting, including recent advances, such as the essential role of *PEG10* in the placental fetal capillaries (with putative collaboration between PEG10 and PEG11/RTL1) (Shiura et al., 2021), the critical roles of PEG11/RTL1 in fetal muscle and brain development (Kitazawa et al., 2020; Kitazawa et al., 2021), and their involvement in various human diseases (Pandya et al., 2021; Whitely et al., 2021; Black, et al., 2023). We also introduce a new line of emerging research, as *PEG10* and *PEG11/RTL1* have also attracted considerable attention of forming virus-like particles (VLPs) (Abed et al., 2019; Segel et al., 2021), in addition to being retrovirus-derived domesticated genes (Kaneko-Ishino and Ishino, 2012; Kaneko-Ishino and Ishino, 2015).

## 2 Discovery of *PEG10* and *PEG11/RTL1* as retrovirus-derived imprinted genes

### 2.1 Discovery and identification of *PEG10* and *PEG11/RTL1*

**FIGURE 1:**
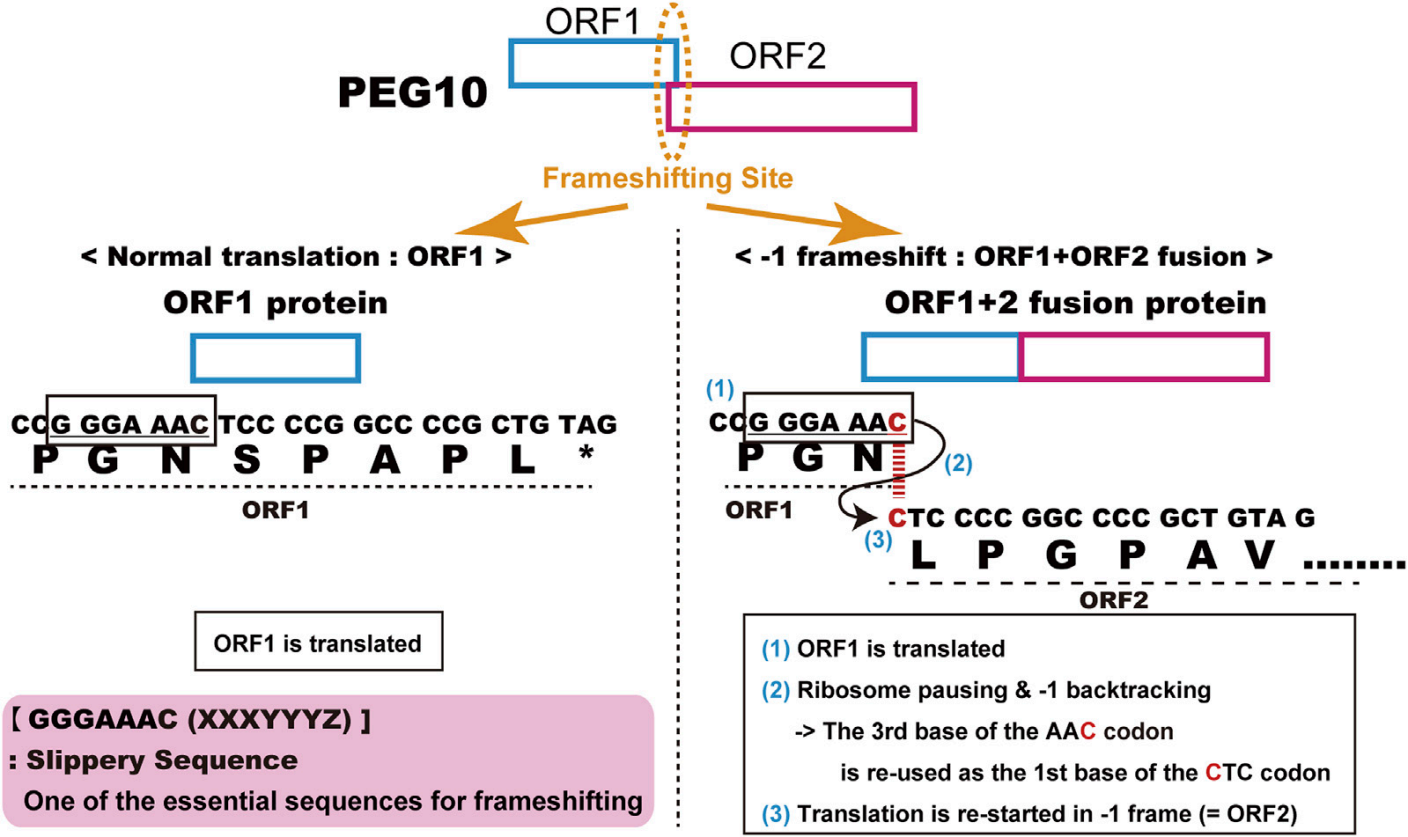
Details of PEG10 frameshifting in mice. *Peg10* produces two different proteins, PEG10-ORF1 by normal translation (left) and the PEG10 ORF1 and ORF2 fusion protein (PEG10-ORF1/2) by using the “−1” translational frameshifting mechanism (right).

The human and mouse form of *PEG10* (Ono et al., 2001) was identified as the 10 th paternally expressed gene in our comprehensive screening of imprinted genes in humans and mice (Kaneko-ishino et al., 1995; Kuroiwa et al., 1996; Kagitani et al., 1997; Miyoshi et al., 1998; Kobayashi et al., 2000; Okutsu et al., 2000; Ono et al., 2001; Ono et al., 2003; Kitazawa et al., 2020). The name “*PEG10”* is used as the official designation, although prior to or around the time of its publication, partial or incomplete *PEG10*-like cDNA sequences had been already discovered and/or reported in some mammalian species, under such designations as TGF1-β1-repressed transcript 1 (TRT1, American mink) (Ralph et al., 1993; Butler et al., 2001), MyEF-3, MyEF3-like (mouse) (Steplewski et al., 1998), Edr (mouse) (Shigemoto et al., 2001) and KIAA1051 (human) (Butler et al., 2001; Volff et al., 2001). Now, they are perceived to be orthologs of or identical to human *PEG10*. *PEG10* has two long open reading frames (ORFs), ORF1 and ORF2, which have high homology to the GAG and POL proteins of the sushi-ichi retrotransposon isolated from pufferfish (Poulter and Butler, 1998), respectively. Like other LTR retrotransposons and retroviruses, *PEG10* produces two different proteins, GAG-like PEG10-ORF1 and GAG-POL like PEG10-ORF1 and ORF2 fusion (PEG10-ORF1/2) proteins. In the normal eukaryotic translation system, the *PEG10* mRNA should only produce the PEG10-ORF1 protein, because its translation is halted at the ORF1 stop codon and the ORF2 open reading frame is in a different frame than ORF1. In fact, it has been shown experimentally that *PEG10* uses the “minus 1” translational frameshifting mechanism to produce PEG10-ORF1/2 protein (Shigemoto et al., 2001). The details of the mechanism are as follows: 1) ORF1 is translated, 2) ribosome pauses and backtracks one place at the highly conserved consensus “slippery sequence”, GGGAAAC, just before the ORF1 stop codon and immediately after the start of ORF2, 3) the ribosome restarts translation using the ORF2 reading frame from the backtracked site, resulting in the production of an ORF1 and ORF2 fusion protein (Figure 1). This evidence strongly supports the notion that *PEG10* is the gene derived from an LTR retrotransposon or retrovirus, although *PEG10* in its current form has lost transpositional activity because it lacks LTR sequences at both ends as well as certain motifs required for retrotransposition, such as reverse transcriptase, RNase H integrase and DNA integrase in POL.

*PEG11/RTL1* was identified as one of the novel imprinted genes in the ovine *callipyge* locus associated with muscle hypertrophy primarily in the hindquarters (Charlier et al., 2001). It was the second such imprinted gene discovered, with approximately 30% homology to the *GAG* and *POL* of the sushi-ichi retrotransposon (Charlier et al., 2001; Brandt et al., 2005; Youngson et al., 2005; Ono et al., 2006). Like *PEG10*, it lacks LTR sequences at both ends and the motifs required for retrotransposition in POL, while unlike *PEG10* it has *GAG-* and *POL-*like sequences in the same ORF, that is, it loses the −1 translational frameshift mechanism. *PEG11/RTL1* was also reported as a retrotransposon-derived fragment, *Hur1,* on human chromosome 14 (Butler et al., 2001). Thus, *PEG10* and *PEG11/RTL1* were identified as paternally expressed imprinted genes with significant homology to the suchi-ichi retrotransposon, but are considered to be of retroviral origin.

### 2.2 *PEG10* causes parthenogenetic death and early embryonic lethality

**FIGURE 2:**
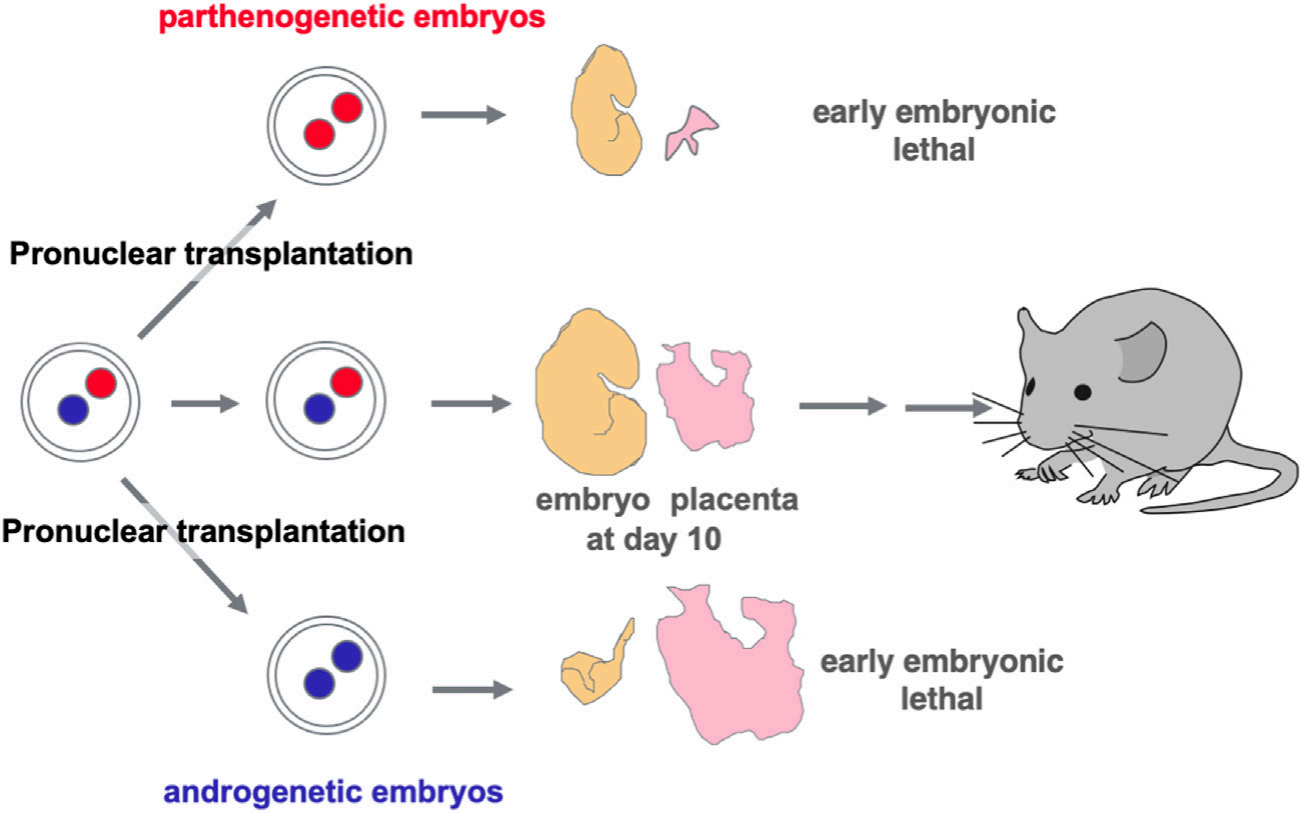
Discovery of genomic imprinting via pronuclear transplantation experiment Both the parthenogenetic and androgenetic embryos produced by pronuclear transplantation experiments exhibit early embryonic lethality with completely different morphological defects at 10.5 dpc. In both cases, placental development was severely disrupted.

**FIGURE 3:**
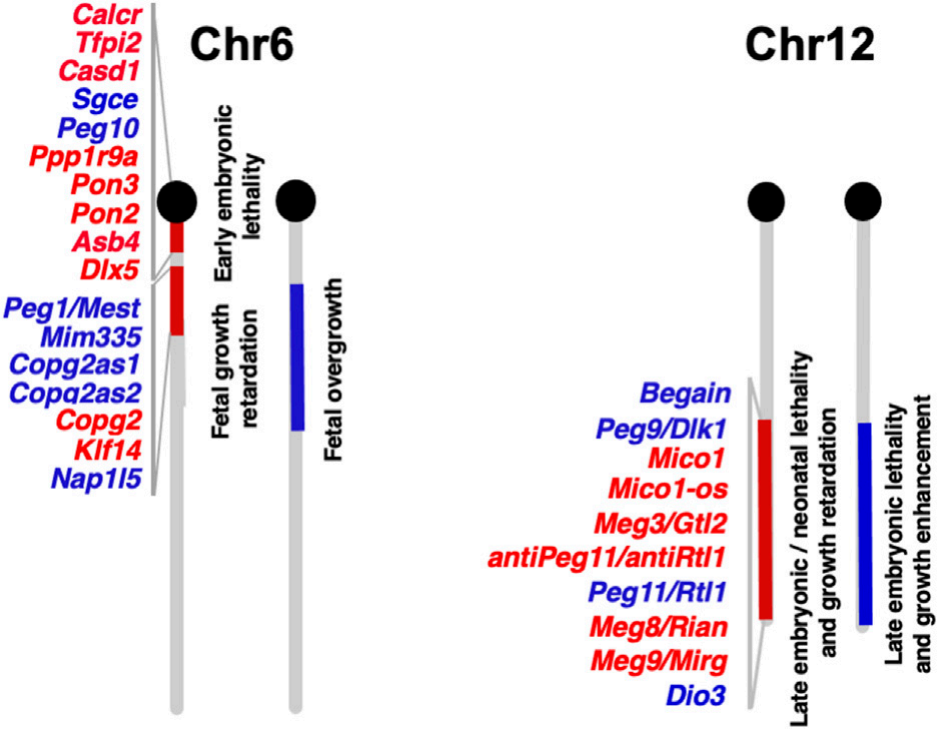
Imprinted genes on mouse chromosomes 6 and 12 The imprinted gene map of mouse chromosomes 6 and 12 where *Peg10* and *Peg11/Rtl1* are localized, respectively. Paternally and maternally expressed genes are shown in blue and red, respectively. The imprinted gene data is from the homepage of the Mary Lyon Center at MRC Harwell (https://www.har.mrc.ac.uk/about-us/history/imprinting-resource/).

Functional differences between paternally and maternally derived genomes and/or chromosomal regions in mammals were originally discovered by pronuclear transplantation experiments (Mann and Lovell-Badge, 1984; McGrath and Solter, 1984; Surani et al., 1984) and by a series of genetic experiments in mice with the Robertsonian translocation (Cattanach and Kirk, 1985; Cattanach and Beechey, 1990). Both parthenogenetic and androgenetic embryos, containing only maternal and paternal genomes, respectively, display early embryonic lethality at 9.5 days post coitus (dpc), demonstrating that both parental genomes are necessary for normal mammalian development (Figure 2). The former exhibit severe placental dysplasia while the embryos themselves are somewhat small but look normal, indicating that the placental defect is the major cause of parthenogenetic death, while the latter exhibit severe embryonic retardation with abnormally large placentas (Mann and Lovell-Badge, 1984; McGrath and Solter, 1984; Surani et al., 1984). Mice with uniparental duplication of a certain specific region showed developmental and growth abnormalities leading to lethality at various embryonic and postnatal stages, are thus designated as imprinted regions (Cattanach and Kirk, 1985; Cattanach and Beechey, 1990). Most of the imprinted genes have been mapped to these imprinted regions with distinct imprinting phenotypes. Mice with maternal duplication of proximal chromosome 6 exhibit early embryonic lethality and this is the only imprinted region that exhibits this result upon maternal duplication (Cattanach and Beechey, 1990) (Figure 3), suggesting that the imprinted gene responsible for the parthenogenetic death, if any, should be located there.

Using data from the Human Genome Project, extensive genome screening was performed around human chromosome 7q21, the region orthologous to mouse proximal chromosome 6. *PEG10* was identified as a paternally expressed gene in both humans and mice (Ono et al., 2001) adjacent to paternally expressed *Sarcoglycan epsilon* (*SGCE*) (Piras et al., 2000). Exogenous DNA-derived imprinted genes, such as retrovirus-derived imprinted genes, were targets of our comprehensive imprinted gene screening. This was due to our hypothesis that genomic imprinting arose as a genome defense mechanism, similar to Barlow’s hypothesis (Barlow, 1993), and the assumption that such inserted retrovirus-derived genes were imprinted. In this imprinted region, only two paternally expressed genes, *PEG10* and *SGCE,* exist together with other maternally expressed genes (Ono et al., 2003) and loss-of-function mutations on *SGCE* are reported to cause myoclonus dystonia syndrome, which is characterized by bilateral, alcohol-sensitive myoclonic jerks mainly affecting the arms and axial muscles (Zimprich et al., 2001), suggesting that retrovirus-derived *PEG10* may be causative gene in placental dysplasia.

**FIGURE 4:**
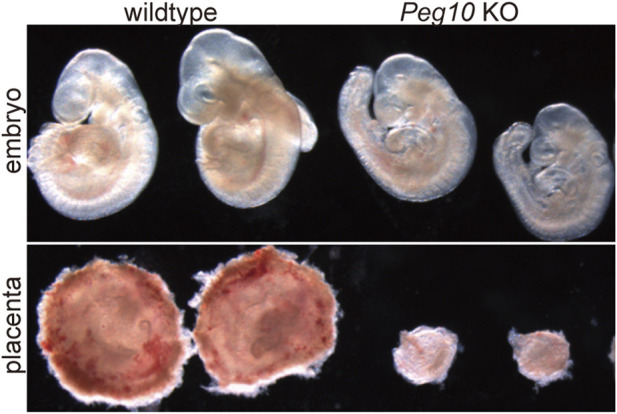
*Peg10* KO mice exhibit severe placental dysplasia at 9.5 dpc *Peg10* KO embryos are morphologically normal in appearance albeit somewhat smaller than the wildtype. However, their placentas exhibit severe dysplasia owing to the loss of both the labyrinth and spongiotrophoblast layers.

As expected, mice with paternally derived *Peg10* KO allele (hereafter referred to as *Peg10* KO mice) exhibited early embryonic lethality, that is, all the *Peg10* KO mice died before 10.5 dpc due to severe placental dysplasia, as did the parthenogenetic embryos (Figure 4) (Ono et al., 2006). The KO placenta almost completely lost two major layers, the labyrinth and spongiotrophoblast layers, demonstrating that *Peg10* is an indispensable gene for early trophoblast growth and differentiation in early placental formation. Thus, *Peg10* is evidently a major causative gene of parthenogenetic death as well as the early embryonic lethal phenotype caused by maternal duplication of proximal chromosome 6 (Ono et al., 2006).

### 2.3 *PEG11/RTL1* causes late embryonic/neonatal lethality as well as placental abnormalities in Kagami-Ogata and Temple syndromes

Mice with paternal and maternal duplication of distal chromosome 12 exhibit late fetal lethality associated with a variety of abnormalities (Figure 3) (Catanach and Beechey, 1990), like mice with paternal and maternal uniparental disomies for chromosome 12 (pUPD12 and mUPD12), respectively (Georgiades et al., 2000): the former exhibit placental overgrowth and malformation, dysplasia of the thoracic ribs, and a larger muscle fiber size as well as prenatal lethality, while the latter exhibit poor placental growth, thinner skeletal muscle fibers and late fetal/neonatal lethality.

Interestingly, both human pUPD14 and mUPD14 patients display symptoms quite similar to the orthologous mouse pUPD12 and mUPD12 cases, respectively (Kotzot, 2004; Kagami et al., 2005). They are respectively designated Kagami-Ogata syndrome (KOS 14) and Temple syndrome (TS14). KOS14 is characterized by neonatal lethality with respiratory failure, placentomegaly, polyhydramnios, developmental delay and/or intellectual disability, and feeding difficulties (Kagami et al., 2005; Kagami et al., 2015). TS14 is characterized by prenatal and postnatal growth retardation, feeding difficulties, muscle hypotonia, motor delay, early onset of puberty, and mild intellectual disability (Ioannides et al., 2014; Kagami et al., 2015).

Multiple imprinted genes have been identified in the large *DLK1-DIO3* imprinted region of over 820 kb (Kobayashi et al., 2000; Miyoshi et al., 2000; Schmidt et al., 2000; Wylie et al., 2000; Charlier et al., 2001; Cavaille et al., 2002; Hernandez et al., 2002; Hernandez et al., 2004; Davis et al., 2005): three paternally expressed genes (*Delta-like 1 homologue* (*DLK1*), *PEG11/RTL1* and *Iodothyronine deiodinase 3* (*DIO3*)) that encode proteins, and four non-coding maternally expressed genes (*Maternally expressed 3* (*Meg3*)*/Gene trap locus 2* (*Gtl2*), *antiPeg11/antiRtl1*, *Meg8/RNA imprinted and accumulated in nucleus* (*Rian*), *Meg9/microRNA containing gene* (*Mirg*)) that produce either microRNAs (miRNAs) or small nucleolar RNAs (snoRNAs). It should be noted that *antiPeg11/Rtl1as* is a miRNA host gene and the miRNAs produced from *antiPeg11/Rtl1as* are involved in *Peg11/Rtl1* mRNA degradation through an RNAi mechanism (Seitz et al., 2003; Davis et al., 2005; Ito et al., 2015).

*Peg11*/*Rtl1* exhibits specific expression in the endothelial cells of the placental fetal capillaries and plays an important role in maintaining normal placental function. In mice, *Peg11/Rtl1* is necessary for the maintenance of the structure and function of the placental fetal capillaries, and both loss and overexpression of *Peg11/Rtl1* can result in developmental abnormalities and/or lethality in the placenta and fetus (Sekita et al., 2008; Kitazawa et al., 2017). Loss of *Peg11/Rtl1* leads to impaired maintenance of the placental fetal capillary structure, resulting in underdeveloped placentas and subsequent fetal growth retardation, ultimately leading to prenatal and postnatal lethality (Sekita et al., 2008; Kitazawa et al., 2017). The endothelial cells are attacked by the surrounding trophoblast cells, so the fetal capillaries become clogged by the invading trophoblast cells (Sekita et al., 2008). Overexpression of *Peg11/Rtl1* causes heavy damage to the surrounding trophoblast cells and causes abnormal morphology of the placental fetal capillary, hindering the formation of normal vascular structures resulting in placentomegaly, like that seen in KOS14 patients caused by paternal duplication of chromosome 14, where *PEG11/RTL1* is located (Kagami et al., 2008; Sekita et al., 2008; Kagami et al., 2015). Deletion of *microRNA-127*, which regulates *Peg11/Rtl1*, also results in elevated *Peg11/Rtl1* expression in the placenta and induces placentomegaly, resembling the phenotype observed with *Peg11/Rtl1* overexpression (Ito et al., 2015). Thus, *PEG11/RTL1* is a major causative gene for the late embryonic/neonatal lethal phenotype caused by paternal and maternal duplications of distal chromosome 12 as well as the placental abnormalities observed in the two human genomic imprinting disorders, KOS14 and TS14 (Kagami et al., 2008; Sekita et al., 2008; Kitazawa et al., 2017). It should be noted that *DLK1* may also be involved in pre- and postnatal growth retardation in TS14 to some extent (Moon et al., 2002; Abdallah et al., 2011).

## 3 *PEG10* and *PEG11/RTL1* in normal development and human disorders

*PEG10* and *PEG11/RTL1* are essential placental genes in eutherian mammals that are associated with early embryonic and late/neonatal lethality in mice, respectively. They also play roles in normal eutherian development and in several human diseases.

### 3.1 *PEG10* has multiple essential roles in the placenta

*PEG10* is essential for the development of several trophoblast cell lines in the placenta. However, how the PEG10 protein regulates trophoblast development has remained largely unreported. One reason for this, as described above, is that its viral-like properties, which are highly conserved among therian mammals: *Peg10* produces not only two proteins, the GAG-like ORF1 protein and the GAG-like ORF1 and POL-like ORF2 fusion protein, but also various PEG10 fragments generated by its self-cleavage activity. It is conceivable that each of the PEG10 proteins and fragments has different functions and confers considerable functional diversity on the *PEG10* gene.

**FIGURE 5:**
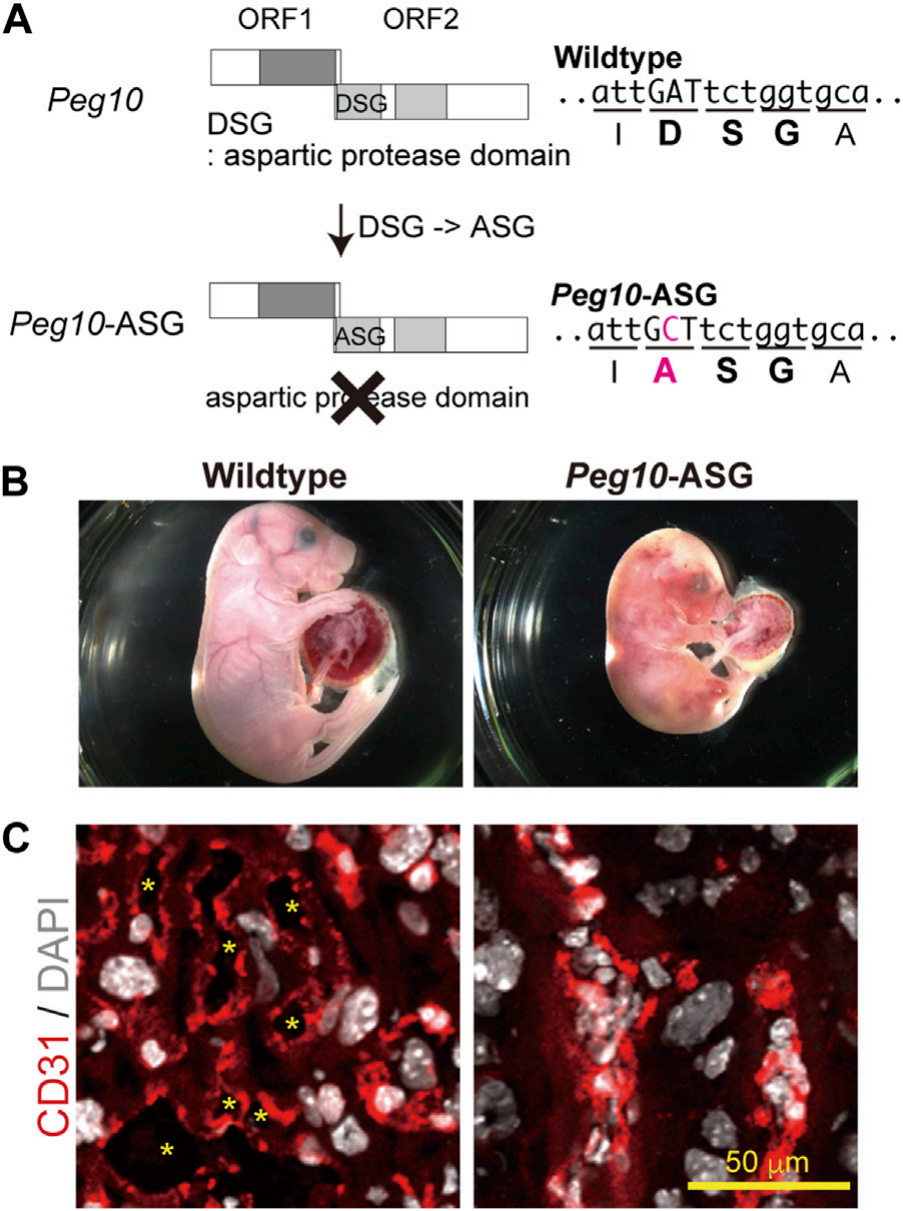
*Peg10*-protease-motif mutant mice (*Peg10*-ASG) exhibit perinatal lethality **(A)** Schematic of the Peg10-ASG mutation. The aspartyl protease domain composed of DSG residues was disrupted by substitution of alanine (A) for the aspartic acid (D). A point mutation, A to C, results in the amino acid substitution, DSG to ASG, in Peg10-ASG mice. **(B)** Wildtype (left) and Peg10-ASG (right) embryos at 18.5 dpc. Approximately 50% of the Peg10-ASG embryos recovered at this stage were already dead. **(C)** Immunofluorescence analysis of the labyrinth layer of placentas at 18.5 dpc with an anti-CD31 antibody (vascular endothelial cells, red). Nuclei were stained with DAPI (white). The inflamed fetal vasculature had become clogged, resulting in impairment of feto-maternal exchange in the Peg10-ASG placental labyrinth layer. The asterisks indicate the fetal blood space. These images have been reproduced from Shiura et al., 2021 under the CC-BY 4.0 permissions protocol (http://creativecommons.org/licenses/by/4.0/).

Based on this concept, it was considered important to elucidate the functions of each of the PEG10 products and motifs by analyzing a series of *Peg10* mutant mice harboring deletion of each PEG10 protein and/or mutations on the conserved motifs. Indeed, *Peg10* protease motif mutant mice unexpectedly exhibit perinatal lethality rather than the early embryonic lethality observed in the *Peg10* KO mice (Figure 5) (Shiura et al., 2021). A point mutation was introduced into the DSG viral aspartic protease motif, which consists of three consecutive amino acids, aspartic acid (D) - serine (S) - glycine(G), in the POL-like ORF2 by replacing catalytic aspartic acid (D) with an alanine (A) residue using the CRISPR-Cas9 system. Such *Peg10*-ASG mutant mice, which lose the self-cleavage activity of PEG10, had an apparently normal appearance until mid-gestation, but then exhibited embryonic and placental growth retardation from around 12.5 dpc, and about half of them died at 18.5 dpc. Severe inflammation was detected around the fetal vasculature in the labyrinth layer of the mutant placenta, suggesting that the perinatal lethality observed in the *Peg10*-ASG mutant is caused by disruption of the feto-maternal circulation (Shiura et al., 2021).

In the labyrinth layer, PEG10 is expressed in the three layers of trophoblast cells, two layers of syncytiotrophoblast cells (SynT-I and II) and one layer of mononucleated sinusoidal trophoblast giant cells (s-TGCs), surrounding the fetal capillary endothelial cells, indicating that PEG10 expression in these is essential for fetal capillary maintenance during mid to late gestation. It is of interest to consider the possibility that PEG10 is required for feto-maternal immunotolerance because PEG10 expressing cells face the maternal blood in the labyrinth layer and therefore might thus protect the fetal capillaries from the maternal immune system. This hypothesis will require further study on how PEG10 specifically works in fetal capillary maintenance, but in any event it is evident that *PEG10* has multiple essential functions in the placenta, and these two functions were crucial to the emergence of the chorio-allantoic placenta in eutherian mammals.

### 3.2 Presumable interaction between *PEG10* and *PEG11/RTL1* in the placenta

**FIGURE 6:**
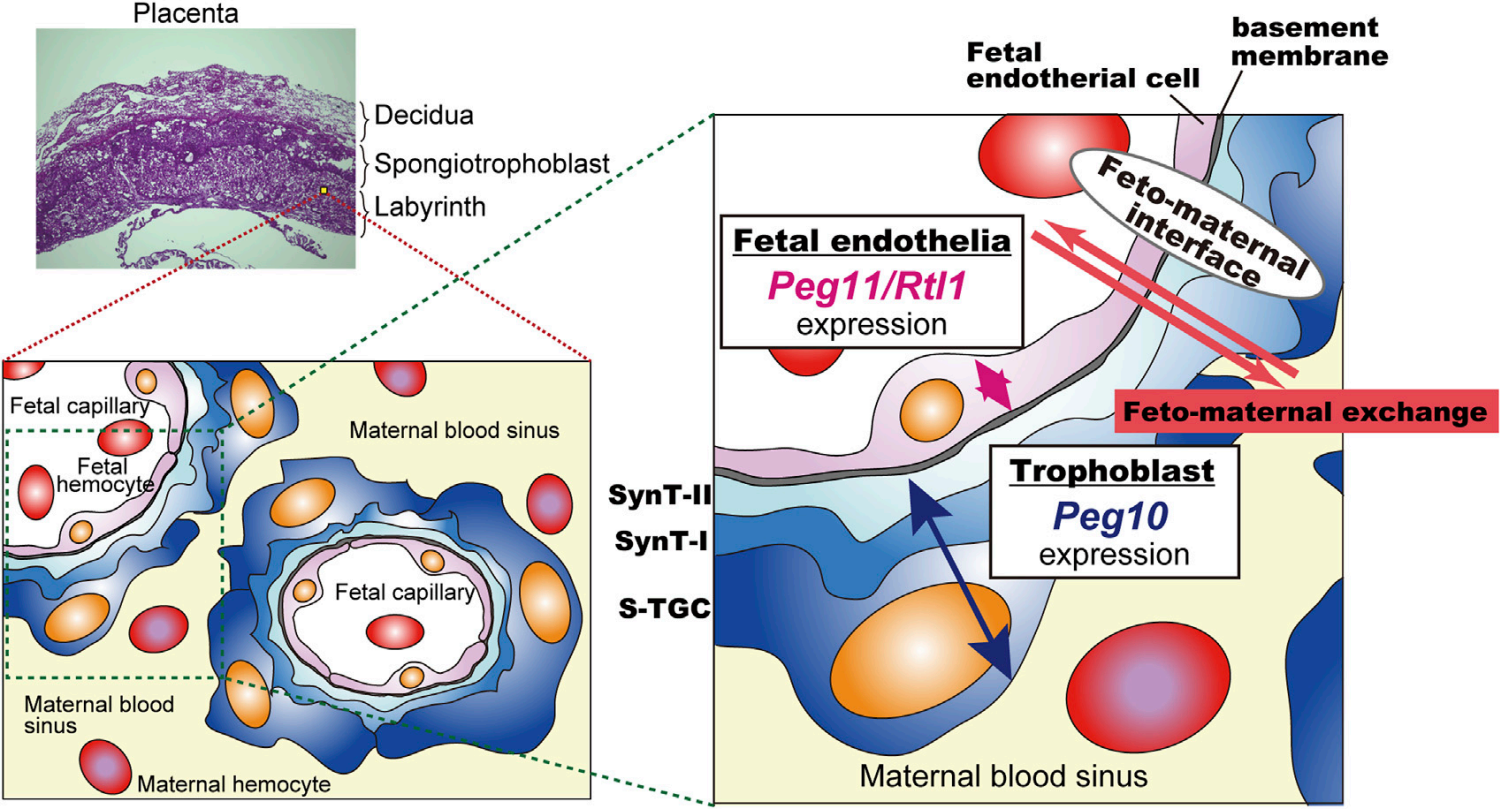
A model for the cooperation between PEG10 and PEG11/RTL1 at the feto-maternal interface The PEG10 and PEG11/RTL1 proteins are expressed and function on one side of the fetal capillaries, in the trophoblast layer or in the fetal endothelial cells, to ensure normal feto-maternal exchange. This figure has been reproduced from Shiura et al., 2021 under the CC-BY 4.0 permissions (http://creativecommons.org/licenses/by/4.0/). Figure 5 and Figure 6 include minor modifications and formatting changes from the original figures.

*Peg10*-ASG mutant mice exhibit perinatal lethality similar to *Peg11/Rtl1* KO mice that exhibit late embryonic lethality due to severe damage of the fetal capillary network during mid-late gestation (Sekita et al., 2008; Kitazawa et al., 2017; Shiura et al., 2021). Both of the PEG10 and PEG11/RTL1 proteins are expressed in the fetal capillaries, but the former is expressed in the trophoblast layers, the SynT-I and II layers and the single s-TGC layer, while PEG11/RTL1 is restricted to endothelial cells, demonstrating that *Peg10* and *Peg11/Rtl1* function in the feto-maternal interface in a face-to-face manner. This suggests that the cooperation of PEG10 and RTL1 provides the essential architecture for the feto-maternal interface in the eutherian placenta so as to maintain a normal fetal vasculature for the exchange of nutrients/waste and O_2_/CO_2_ gas between the fetal and maternal blood (Figure 6).

After 15.5 dpc, the PEG10 protein signals became prominent in s-TGCs facing the maternal blood sinus, strongly suggesting that the trophoblast cells in the fetal capillaries, especially s-TGCs, afford some defensive protection to the fetal endothelial cells against certain hazardous events. One possibility is that PEG10 protease activity is required for immunotolerance between the mother and embryos. The PEG10 protease may induce an aggressiveness in trophoblasts toward maternal immune cells, then RTL1 would play a defensive role in fetal capillary endothelial cells against an attack by the PEG10-expressing trophoblast cells. Recent phylogenic analysis predicts that the mouse and human-type hemochorial placenta is the ancestral eutherian placenta with a feto-maternal interface in which the trophoblast surface has direct contact with the maternal blood (Wildman et al., 2006; Roberts et al., 2016). Therefore, it is highly likely that the domestication of *PEG10* and *RTL1*, before and after the divergence of the eutherians and marsupials, respectively, must have been critical events and exerted a driving force in the evolution of the eutherian viviparous reproductive system.

### 3.3 Essential placental roles of *PEG11/RTL1* in other eutherian species

Abnormal expression of *PEG11/RTL1* in humans has been associated with intrauterine growth restriction (IUGR), fetal anomalies, and death (Sgardioli et al., 2013; Prats-Puig et al., 2017; Fujioka et al., 2019). This placental insufficiency may be a cause of IUGR and hydrops symptoms reported in other species as well. These findings suggest that PEG11/RTL1 plays an important role in placental function in these other species via fetal capillary maintenance and/or placental angiogenesis.

In horses (*Equus caballus*), *PEG11/RTL1* is paternally expressed and localized within the endothelial cells of the equine chorioallantois (Dini et al., 2021). Knockdown of *PEG11/RTL1* in cultured primary endothelial cells from the equine placental microvasculature resulted in the loss of sprouting ability, which is a fundamental process in neovascularization, further confirming the association between equine *PEG11/RTL1* expression and endothelial cells and its importance in placental angiogenesis (Dini et al., 2021). Additionally, an association between abnormal expression of *PEG11/RTL1* and the development of hydrallantois has been demonstrated (Dini et al., 2021). This suggests that *PEG11/RTL1* is also essential for placental angiogenesis, and that its abnormal expression could lead to placental dysfunction.

*PEG11/RTL1* is the most frequently abnormally expressed gene among all of the abnormally expressed imprinted genes in cloned animals such as pigs and cows, consistently associated with post-implantation pregnancy loss through unknown mechanisms (Yu et al., 2018). The majority of these clones display abnormalities in placental formation, including disruption of the placental vascular system and hydrops-like conditions, once again suggesting the essential role of normal *PEG11/RTL1* expression in the placenta in eutherians.

### 3.4 *PEG11/RTL1* in muscle development

The activity of *PEG11/RTL1* in muscle is well known from studies on *Callipyge* sheep that exhibit a muscle hypertrophy phenotype (Charlier et al., 2001; Fleming-Waddell et al., 2009; Byrne et al., 2010). The callipyge phenotype is caused by the CLPG mutation, which is an A to G transition in a highly conserved dodecamer motif. This motif is situated in the 90-Kb intergenic region that separates the *DLK1* and *MEG3/GTL2* genes in the imprinted domain (Freking et al., 2002; Smit et al., 2003). The CLPG mutation is thought to inactivate a muscle-specific silencer, thereby causing prolonged, ectopic expression of *DLK1-DIO3* region genes in postnatal skeletal muscle. *DLK1* is critically involved in muscle development and is therefore implicated in the muscular hypertrophy in sheep as well as a mouse model of muscle hypertrophy (Davis et al., 2005; Abdallah et al., 2011). In addition to *DLK1,* it is highly likely that *PEG11/RTL1* is also responsible for the muscular hypertrophy of callipyge sheep (Xu et al., 2015). In sheep, the expression of *PEG11/RTL1* is relatively high up to the late fetal stage, decreases from just before birth, and is hardly expressed after birth. In contrast, in callipyge sheep, *PEG11/RTL1* expression decreases once just before birth, and increases again after birth. *PEG11/RTL1* expression in the semimembranosus (SM) skeletal muscle exhibited a 45-fold increment compared to wild type sheep (Byrne et al., 2010) and exhibited a full hypertrophy phenotype at 2 weeks of age (Carpenter et al., 1996; Jackson et al., 1997; Freking et al., 1998). Thus, the callipyge mutation recapitulates the normal fetal-like *PEG11/RTL1* expression program during postnatal development and this may contribute to the emergence of the muscle hypertrophy phenotype (Byrne et al., 2010), suggesting that abnormal *PEG11/RTL1* overexpression is required for the callipyge phenotype.

Additionally, ectopic expression of ovine *PEG11/RTL1* causes muscle hypertrophy in mice, mimicking the ovine callipyge phenotype (Xu et al., 2015). In experiments with transgenic (Tg) mice that overexpressed ovine *PEG11/RTL1* with type IIB fast twitch muscle fibers which were predominantly affected in *Callipyge* sheep before and after birth, the size of the extensor digitorum longus (EDL) that is rich in type IIB fast twitch muscle fibers increased in +/Tp and Tp/Tp (15.7% and 24.6%, respectively) compared with WT mice. This implies that the EDL muscle myofibers are enlarged by overexpression of *PEG11/RTL1* (Xu et al., 2015).

**FIGURE 7:**
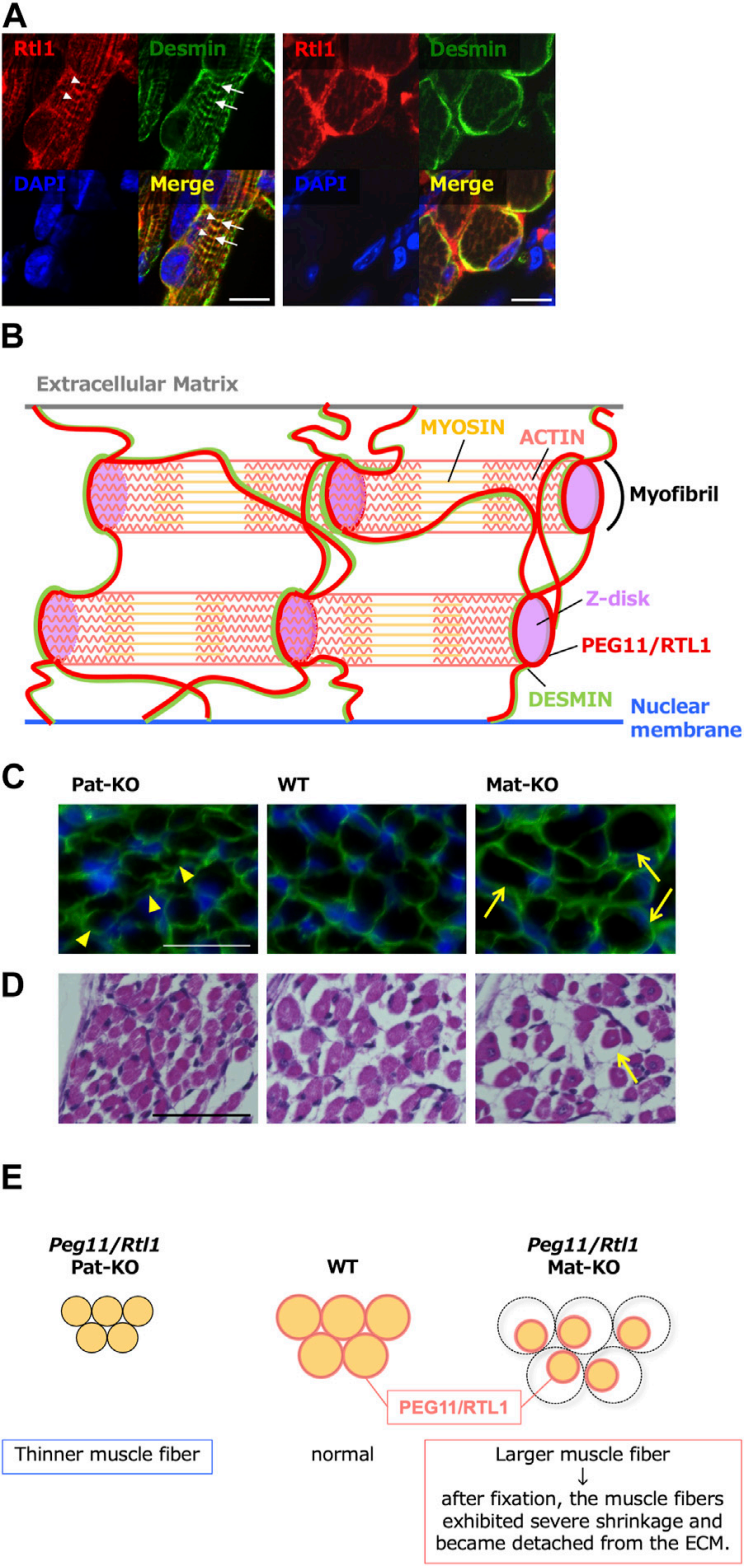
Role of PEG11/RTL1 in muscle fibers, presumably involving DESMIN **(A)** Immunofluorescence staining of the PEG11/RTL1 protein in the neonatal forelimb muscles. Long axis views (left) and cross-sectional views (right) of the muscle fibers. Co-immunostaining with PEG11/RTL1 (red; arrowheads), DESMIN (green; arrows) and DAPI (blue), and their merged images. PEG11/RTL1 is closely located to DESMIN at the level of the Z-disc. Scale bars: 20 μm. **(B)** Schematic diagram of the interaction between PEG11/RTL1 and DESMIN (hypothesis). By colocalizing with DESMIN, the PEG11/RTL1 protein plays specific roles in the function of the fetal/neonatal muscle fibers, such as stabilizing the muscle contractile apparatus and/or regulating muscle constriction with DESMIN. **(C)** Co-immunostaining with laminin (green) and DAPI (blue) (top row). The arrowheads in the Pat-KO column indicate thinner muscle fibers and the arrows in the Mat-KO column indicate large muscle fibers. The neonates were not fixed before being embedded in OCT compound. **(D)** HE staining of neonate intercostal muscle: Pat-KO (left), wild type (middle) and Mat-KO (right). The arrow in the Mat-KO column indicates muscle fibers that have severe shrinkage and became detached from the ECM. Scale bars: 50 μm. Neonates were fixed in Super Fix. **(E)** Schematic diagram of PEG11/RTL1 expression and structural abnormalities in neonatal skeletal muscles in Pat- and Mat-KO mice. The muscle fibers lacking PEG11/RTL1 expression in Pat-KO become thinner (left) compared to the WT (middle). On the other hand, the muscle fibers of Mat-KO with PEG11/RTL1 overexpression appear thicker, but when the tissue was fixed, abnormal contraction of the muscle fibers occurred and detachment from the ECM was observed (right). Images of A, C and D have been reproduced from Kitazawa et al., 2020 under the CC-BY 4.0 permissions (http://creativecommons.org/licenses/by/4.0/).

In mouse skeletal muscle, expression of *Peg11/Rtl1* was detected in the late fetal and neonatal stages but gradually decreased soon after birth and disappeared almost completely at around 2 weeks after birth (Kitazawa et al., 2020). *Peg11/Rtl1* deficient mice exhibited significantly thinner muscle fibers. While *Peg11/Rtl1* overexpressed mice exhibited significantly larger muscle fiber sizes, consistent with the CLPG mutation and symptoms in Tg mice (Figure 7). However, after fixation, the muscle fibers of the *Peg11/Rtl1* overexpressed mice exhibited severe shrinkage and became detached from the extracellular matrix (ECM) muscles, indicating this muscle to be immature and more fragile than normal muscle. This is consistent with the TS14 and KOS 14 patients with muscle-related defects, such as feeding difficulties, muscle hypotonia in the former (Ioannides et al., 2014; Kagami et al., 2015) and respiratory failure and feeding difficulties in the latter (Kagami et al., 2005; Kagami et al., 2015).

*In vitro* experiments with cultured cells from *Peg11/Rtl1* deficient and overexpressed mice also showed that PEG11/RTL1 affected the proliferation of satellite cells (SCs) and the structural strength of SC-differentiated myoblasts (Kitazawa et al., 2020). *Peg11/Rtl1* deficient SCs exhibited a 1.5-fold increase in proliferation compared to WT cells, whereas *Peg11/Rtl1* overexpressed SCs displayed a 0.8-fold decrease in proliferation rate. This result indicates that PEG11/RTL1 plays a suppressive role in SC proliferation and that its expression is required for normal proliferation. Moreover, myoblasts differentiated from *Peg11/Rtl1* deficient and overexpressed SCs clearly exhibited a weak or low structural strength, as some of the myoblasts detached from the culture dish and displayed a rounded shape.

In myocytes, PEG11/RTL1 is partially merged with DESMIN, a component of the sarcomere cytoskeleton that links the sarcomere and its membranes (the sarcolemma and nuclear membranes) at the Z-disc (Kitazawa et al., 2020). It acts as a force-generating machinery in the muscle, suggesting that PEG11/RTL1 plays a certain role in the fetal/neonatal muscle fibers, such as stabilizing the muscle contractile apparatus and/or regulating muscle contraction before birth. The weak muscles and reduced movement of the fetuses may be an adaptation to the eutherian viviparous reproduction system with a long gestation period, as ensuring a safe pregnancy is advantageous to both the mother and fetus. Therefore, it is of interest to address the question whether PEG11/RLT1 is responsible for such adaptive regulation. This is because PEG11/RTL1 presumably must have critically contributed to the prolonged gestation period in eutherians by maintaining the proper functioning of the fetal capillary network over time (Sekita et al., 2008; Kitazawa et al., 2017). Human babies and mouse pups need maternal care long after birth, even though most eutherian pups become relatively independent and mobile soon after birth. Therefore, it is also of interest to determine whether postnatal PEG11/RTl1 expression correlates with postnatal locomotive performance in various eutherian species.

### 3.5 *PEG10* and *PEG11/RTL1* in neurological disorders

Recently, an involvement of PEG10 in certain neurological disorders has been reported (Pandya et al., 2021; Black et al., 2023). Pandya et al. demonstrated that PEG10 is a target of UBE3A, the loss of which causes Angelman syndrome (AS), a severe neurodevelopmental disorder, and that it may be involved in the pathophysiology of AS (Pandya et al., 2021). *UBE3A* is a maternally expressed gene, so the loss of maternal *UBE3A* in neurons due to mutation(s) or paternal uniparental disomy of chromosome 15 causes AS, which is characterized by delayed development, intellectual disability, severe speech impairment, ataxia and additional abnormalities (Nicholls and Knepper, 2001; Buiting et al., 2016). However, how UBE3A contributes to the pathophysiology remains elusive. As *UBE3A* encodes an E3 ubiquitin-protein ligase, part of the ubiquitin protein degradation system, they hypothesized that neuronal protein substrates of UBE3A should be elevated in AS neurons, and then identified PEG10 as one of these proteins by assays with neurons differentiated from human-induced pluripotent stem cells (hiPSC) derived from AS patients. They also demonstrated that the regulation of PEG10 by UBE3A is not at the mRNA but protein level and specific to the PEG10-ORF1/2 fusion protein, while PEG10-ORF1 is largely unaffected. Importantly, the change in the transcriptome of hiPSC-derived AS neurons by PEG10 downregulation was exceptionally similar to that by UBE3A reinstatement and both an elevated PEG10-ORF1/2 fusion protein and absence of UBE3A were observed in the neurons in brain samples from *postmortem* AS patients. These results suggest that *PEG10* could well be critically involved in AS pathophysiology. This notion is supported by their finding that *Peg10* overexpression in mouse neuronal precursors resulted in severely impaired migration, which may in turn have caused neuronal dysfunction. Interestingly, they found that PEG10 localized to stress granules (SGs), which is one of the membrane-less organelles composed of proteins and RNAs and is transiently formed in cytosol under stressed conditions. Future study will be required to determine whether the special properties of PEG10, localization to SGs, and secretion in extracellular vesicles, are pathophysiology of AS.

PEG10 is also reported to be associated with the other neurological disorders, including Amyotrophic Lateral Sclerosis (ALS). ALS is a fatal neurodegenerative disease that typically presents in mid-life and is characterized by a progressive loss of motor function (Brown and Al-Chalabi, 2017). Approximately 10% of ALS cases are the familial form of ALS (fALS) and *UBQLN2*, a member of the ubiquilin family implicated in proteasomal degradation, is known as one of the genes responsible for fALS (Deng et al., 2011). Whiteley and others demonstrated that UBQLN2 facilitates PEG10-ORF1/2 protein degradation and much of it is proteasome-dependent in cells, and the PEG10-ORF1/2 protein is specifically upregulated in the spinal cord of ALS patients compared to healthy controls (Whiteley et al., 2021; Black et al., 2023). In addition, a nuclear-localized PEG10 fragment excised by its self-cleavage activity harbored in the pol-like domain of the ORF1/2 protein induces changes in gene expression involved in axon remodeling. These results suggest that PEG10-ORF1/2 accumulation is a major contributor to ALS disease progression. Intriguingly, UBQLN2 is known as a marker protein of SGs, where PEG10 localizes under stress conditions, as observed in the AS study described above (Pandya et al., 2021). Therefore, it is possible that the pathogenic mechanism of AS and ALS overlaps in part and that PEG10 accumulation may be involved in other, as yet unidentified neurological disorders as well.

As already mentioned, *RTL1* is one of the major causative genes for imprinting diseases, KOS14 and TS14, which are caused by abnormal regulation of the imprinting region on human chromosome 14 (Kotzot, 2004; Kagami et al., 2005; Ioannides et al., 2014; Kagami et al., 2008; Kagami et al. 2015). KOS14 and TS14 patients exhibit certain neurodevelopmental symptoms, such as developmental delay and/or intellectual disability as well as feeding difficulties in the former (Kagami et al., 2005; Kagami et al., 2015) and feeding difficulties, motor delay, early onset of puberty and mild intellectual disability in the latter (Ioannides et al., 2014; Kagami et al., 2015).

**FIGURE 8:**
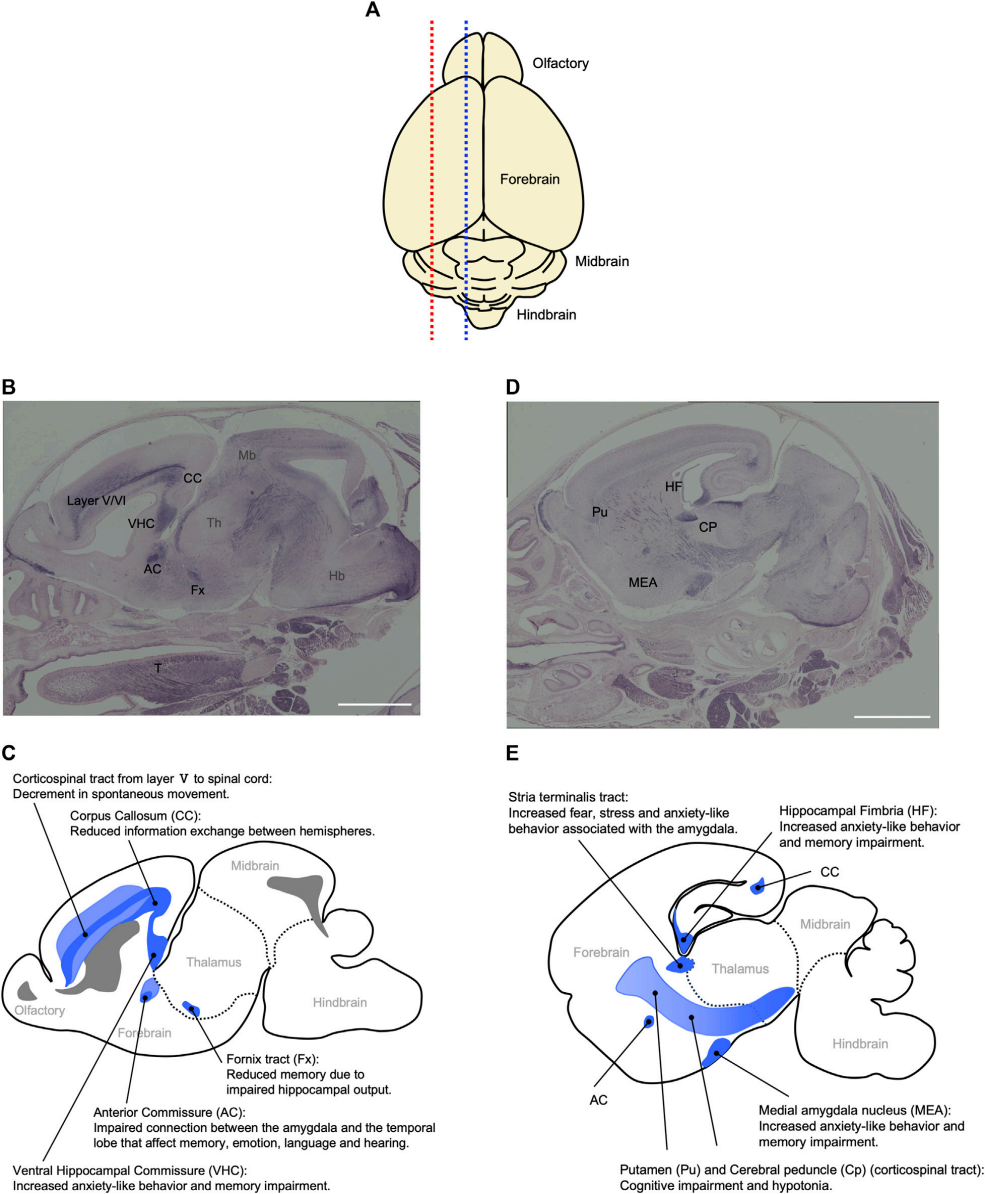
Relationship between behavioral abnormalities in Pat- and Mat-KO mice and PEG11/RTL1 expression sites in mouse neonatal brain. **(A)** Schematic diagram of the mouse P0 neonatal brain. Blue and red dotted lines correspond to **(B, C)** and **(D, E)**, respectively. **(B–E)** Sagittal sections of the P0 neonatal brain **(B, D)** and their corresponding schematic diagrams **(C, E)** that represent the relationship between behavioral abnormalities in Pat- and Mat-KO mice and PEG11/RTL1 expression sites in the mouse neonatal brain. AC: anterior commissure, CC: corpus callosum, CP: cerebral peduncle, Fx: fornix tract, Hb: hindbrain, HF: hippocampal fimbria, MEA: Medial amygdala nucleus, Mb: midbrain, Pu: putamen, T: tongue, VHC: ventral hippocampal commissure, layer V/VI: the fifth and sixth layers of cerebral cortex. Scale bars: 1 mm. The images of **(B, D)** have been reproduced from Kitazawa et al. (2021) under the CC-BY-NC-ND 4.0 permissions (http://creativecommons.org/licenses/by/4.0/).

*Peg11/Rtl1* mRNA is expressed in the central nervus system from the fetal to neonatal periods in mice. The RTL1 protein is detected in the descending tracts, including the corticospinal tract, commissural fibers including the hippocampal commissure, corpus callosum as well as regions of the limbic system, such as the hippocampal fimbria, fornix and medial amygdala nucleus (Figure 8) (Kitazawa et al., 2021). Interestingly, the corticospinal tract and hippocampal commissure are mammalian-specific brain structures. The former runs from layer V of the neocortex to the brainstem and spinal cord and is responsible for fine voluntary skilled muscle movement of the limbs, while the latter is involved in hippocampus-dependent memory output (Abolitz and Montiel, 2003; Mihrshahi, 2006; Kitazawa et al., 2021). In addition, the corpus callosum, which is responsible for communication between the two hemispheres, enabling transmission, integration and separation of information from both sides of the brain, is a eutherian-specific brain structure (Mihrshahi, 2006; Fame et al., 2011; Suárez and Richards, 2014). These results suggest that *PEG11/RTL1* was deeply involved in the functional evolution of the eutherian brain. A high level of PEG11/RTL1 expression was also detected in the locus coeruleus (LC), and *Peg11*/*Rtl1*-deficient mice reportedly exhibit decreased neuronal excitability along with increased delay in the onset of action potentials and inward currents in LC neurons (Chou et al., 2022).

Comprehensive behavioral analyses of the mice with deficiency or overexpression of PEG11/RTL1 support its important neurophysiological roles. These mice exhibit decreased spontaneous movement, increased anxiety-like behavior, and learning and memory impairments (Kitazawa et al., 2021), consistent with the finding that *Peg11/Rtl1* regulates the excitability of LC neurons (Chou et al., 2022). The decrease in spontaneous movement also suggests impairment of the corticospinal tract involved in movement of the trunk and limbs. The increased anxiety-like behavior and memory impairment are thought to be related to the expression of PEG11/RTL1 in the corpus callosum, hippocampal commissure and medial amygdala nucleus. These results are consistent with the neurobehavioral symptoms observed in KOS14 and TS14, such as developmental delay and intellectual disability (Kitazawa et al., 2021). It should be noted that *DLK1* is critically involved in the early onset of puberty in TS14 (Dauber et al., 2017; Macedo and Kaiser, 2019), and that seven microRNAs (miRNAs) are expressed from the maternally expressed *antiPeg11/antiRtl1* and regulate the *Peg11/Rtl1* mRNA level via an RNAi mechanism (Lin et al., 2003; Seitz et al., 2003; Davis et al., 2005). Thus, *DLK1*, *PEG11/RTL1* and *antiPEG11/antiRTL1* have been confirmed to play a major role in a variety of imprinting phenotypes described in KOS14 and TS14 in the placenta, muscle and brain (Kagami et al., 2008; Sekita et al., 2008; Kitazawa et al., 2017; Kitazawa et al., 2020; Kitazawa et al., 2021).

It is reported that *Peg11/Rtl1* is paternally expressed in the subcortex, brain stem and suprachiasmatic nucleus (SCN) while it is maternally expressed in the olfactory bulb, cortex and cerebellum, retina, motor cortex and visual cortex, suggesting that the imprinting status of *Peg11/Rtl1* is brain-region-specific (Hsu et al., 2018). However, it should be noted that the regions where *Peg11/Rtl1* is most highly expressed exhibits paternal expression.

### 3.6 *PEG10* and *PEG11/RTL1* in cancer

There are commonalities between placental development and tumor formation. Understanding these mechanisms should provide a basis for discovering new molecular targets for effective cancer immunotherapy, and *PEG10* and *RTL1* are examples of how this might work. Both *PEG10* and *RTL1* are considered to act as a driving factor in certain types of cancer. There are many reports describing the relationship between PEG10 and cancer, and *PEG10* is seen as an oncogene implicated in the proliferation, apoptosis and metastasis of tumors. Such *PEG10* investigations related to cancer have already been summarized elsewhere (Xie et al., 2018).

It has been reported that overexpression of *PEG11/RTL1* leads to the development of hepatocellular carcinoma (HCC) in mice (Riordan et al., 2013) and that upregulation of *PEG11/RTL1* occurs in a considerable proportion of human HCC samples (Riordan et al., 2013). *RTL1* activation has been implicated as a driver of liver cancer in all Sleeping Beauty (SB)-induced tumors incorporating the Dlk1-Dio3 locus (Hou et al., 2015). Highly enriched *PEG11/RTL1* was also detected in melanoma tissue, especially in early and lateral growth and melanoma cells *in vitro* via activation of the Wnt/β-catenin signaling pathway. Knockdown of *RTL1* in these melanoma cells resulted in suppression of cell proliferation (Fan et al., 2017). PEG11/RTL1 expression levels are significantly higher in breast cancer tissues, and higher levels of RTL1 expression on the cell surface were observed in more invasive breast cancer cell lines. Differential expression of PEG11/RTL1 in breast cancer tissues compared to normal breast tissue has been associated with higher malignancy and vascular infiltration (Mahmoudi et al., 2021). These data suggest that *PEG11/RTL1* may function as a promoter in at least some cancers.

## 4 Domestication of *PEG10* and *PEG11/RTL1* in the mammalian genome

### 4.1 *PEG10* and *PEG11/RTL1* in the evolution of genomic imprinting in mammals

**FIGURE 9:**
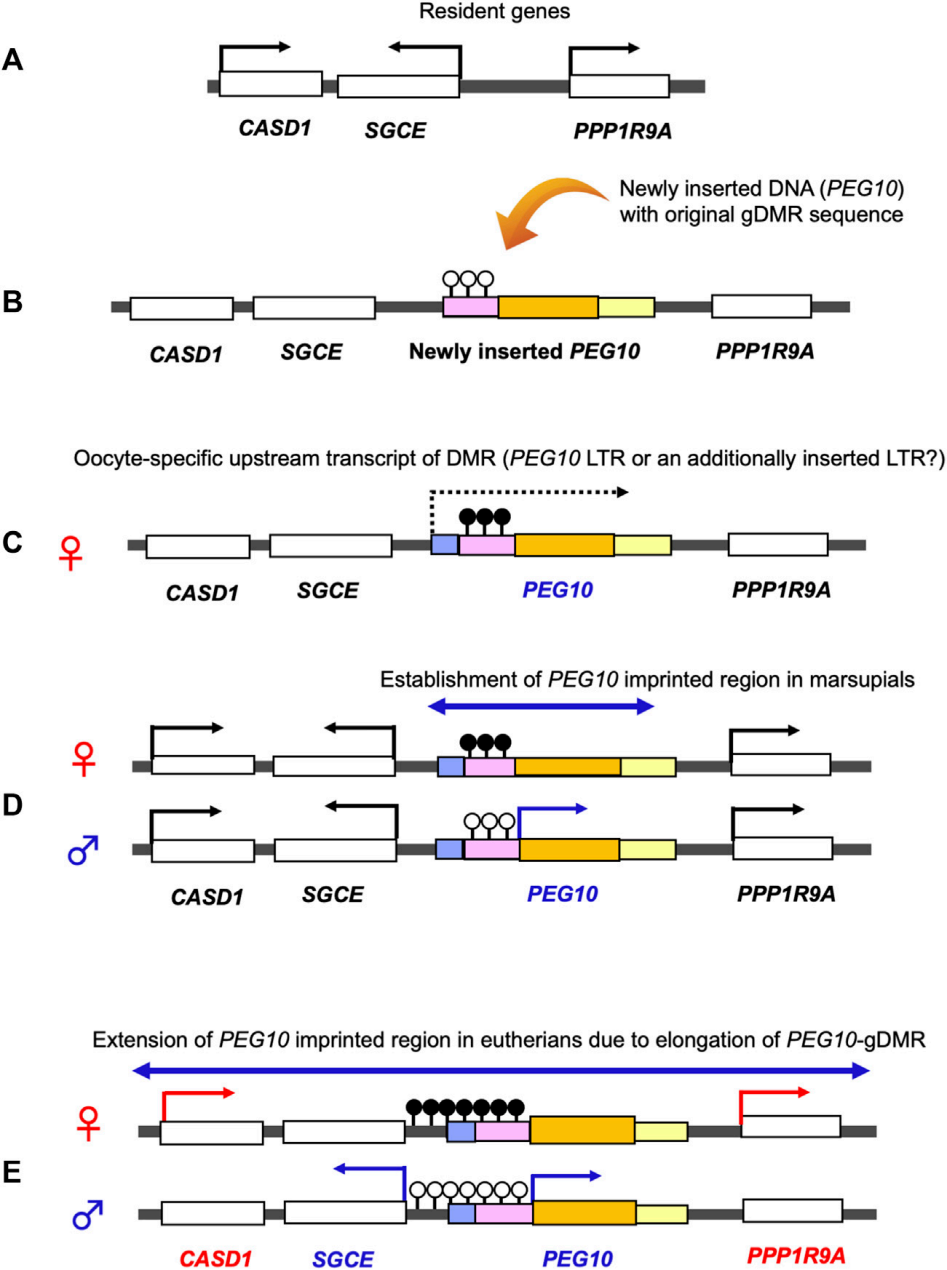
Emergence of the *PEG10* imprinted region. Generation of the PEG10 imprinted region. **(A)** Before an insertion event. A chromosomal region comprises the non-imprinted resident genes *CASD1*, *SGCE* and *PPP1R9A*. **(B)** Insertion of the original *PEG10* retroviral DNA (yellow) containing a *PEG10* promoter as a critical cis-element (pink) and a PEG10 coding sequence (orange). **(C)** DNA methylation on the *PEG10* promoter in the oocyte. Emergence of an upstream promotor (light blue) that expresses an oocyte-specific alternative transcript (dashed line). This transcript goes through the promoter (pink), leading to DNA methylation on the *PEG10* promoter in an oocyte-specific manner. **(D)** Expression of *PEG10* in the marsupial case: *PEG10* is expressed from the paternal allele of a novel imprinted region (arrow), while *SGCE*, *CASD1* and *PPP1R9A* are expressed biallelically (not imprinted). **(E)** Expression of *PEG10* in the eutherian case: *PEG10* and *SGCE* are expressed from the paternal allele due to expansion of the *PEG10*-DMR to the *SGCE* promoter region, while *CASD1* and *PPP1R9A* are maternally expressed via an unknown mechanism.

*PEG10* and *PEG11/RTL1* are paternally expressed genes, the former exists in the maternally imprinted domain where germline DMR (gDMR) is established in the female germline while the latter is in the paternally imprinted domain where gDMR is established in the male germline. *PEG10* has provided a key information on the emergence of canonical genomic imprinting mechanism. Utilizing comparative genome analysis of marsupials in Australia and South America and other eutherians, we demonstrated that domestication of *PEG10* and establishment of *PEG10-*gDMR in the promoter region accompanied by paternal expression occurred simultaneously, before the divergence of the eutherians and marsupials (Suzuki et al., 2007). In the marsupial lineage, only *PEG10* is imprinted under the regulation of *PEG10*-gDMR, suggesting that a large *PEG10* imprinted domain, including *SGCE* and other maternally expressed genes, emerged by extending the *PEG10*-gDMR to the *SGCE* promoter region in the eutherian lineage (Suzuki et al., 2007). This provides clear evidence that newly integrated DNA became the gDMR (Figure 9).

*PEG3* and *PEG5/NNAT* are also of interest in this regard, because these two are paternally expressed imprinted genes (Kuroiwa et al., 1996; Kagitani et al., 1997; Kikyo et al., 1997) that are associated with their promoter gDMRs established in the female germline (Li et al., 2000; Evans et al., 2001; John et al., 2001) and eutherian-specific genes (Kuroiwa et al., 1996; Evans et al., 2005; Suzuki et al., 2011). *PEG3* encodes an unusual zinc finger protein with 11 widely spaced C2H2 type motifs having unique amino acid sequences, but no orthologs exist in marsupials or monotremes (Kuroiwa et al., 1996; Renfree et al., 2011; Suzuki et al., 2011). Its extreme N-terminal region exhibits homology to the GAG protein belonging to the Scan family (Campillos et al., 2006) that first emerged in reptiles and then expanded in the eutherian lineages. More than 50 members of the Scan family genes exist in humans but only *PEG3* is imprinted. The promoter region of *PEG3*-gDMR regulates a 500 kb imprinted domain that includes 7 paternally and maternally expressed genes (He and Kim, 2014). In contrast, *PEG5/NNAT* (Kagitani et al., 1997; Kikyo et al., 1997) is a single imprinted gene. Although *PEG5/NNAT* together with its promoter gDMR are inserted within an intron of the Bladder cancer associated protein (*BCAP*) gene, *BCAP* is not imprinted (Evans et al., 2001; John et al., 2001). *PEG5/NNAT* encodes a proteolipid of unknown origin and is a eutherian-specific gene (Evans et al., 2005). It should be noted that several retrotransposed genes became imprinted genes in their promoter gDMRs (Wood et al., 2007). All of these examples together with *PEG10* provide robust evidence that canonical genomic imprinting regions are associated with the insertion of new genes together with promoter gDMRs into these loci. Further genome analyses demonstrated that all of the gDMRs in the canonical imprinted domains emerged when the imprinting mechanism of these loci first started in the course of mammalian evolution (Suzuki et al., 2011; Renfree et al., 2013; Kaneko-Ishino and Ishino, 2015; 2019; 2022), consistent with the finding that the promoter regions in the canonical maternally imprinted domains became gDMRs (Bartlomei and Ferguson-Simth, 2011). Therefore, it is highly likely that the silencing mechanism of exogenous DNA insertion drove the emergence and evolution of imprinted gene regulation (Kaneko-ishino et al., 2003; Suzuki et al., 2007; Renfree et al., 2013; Kaneko-Ishino and Ishino, 2015; Kaneko-Ishino and Ishino, 2019; Kaneko-Ishino and Ishino, 2022). All of this evidence provides powerful support for the hypothesis that genomic imprinting arose as a defense mechanism against the insertion of exogenous DNA including retroviral insertions (Barlow, 1993; Kaneko-Ishino et al., 2003; Kaneko-Ishino and Ishino, 2015; Kaneko-Ishino and Ishino, 2019; Kaneko-Ishino and Ishino, 2022).

In the case of *PEG11/RTL1*, the intergenic (IG) gDMR located between *DLK1* and *MEG3/GTL2* regulates a large imprinted domain encompassing 820 kb from *DLK1* to *DIO3*. This paternally methylated IG-gDMR is located more than 50 kb away from *PEG11/RTL1,* so it would seem to be unrelated to the domestication of *PEG11/RTL1*. However, all of the imprinted genes between *DLK1* and *DIO3* are eutherian-specific, and IG-gDMR also emerged in eutherians, because there are no orthologous genes in the wallaby and opossum (marsupials) or platypus (monotremes) genomes (Edwards et al., 2008). Therefore, it seems likely that the IG-gDMR emerged with the insertion of *MEG3/GTL2* next to *DLK1*.

There are only two and three paternally imprinted domains in humans and mice, respectively. The *H19*-gDMR in the *H19*-*IGF2* imprinted domain is conserved in both marsupials and eutherians (O’Neill et al., 2000; Smits, et al., 2008), like the *PEG10* imprinted domain (Suzuki et al., 2007). It is likely that *H19* together with *H19*-gDMR were inserted neighboring to *IGF2* in a common therian ancestor. The *PEG10* and *IGF2*-*H19* imprinted regions are only two conserved imprinted domains between marsupials and eutherians that have the apparent and conserved gDMRs, so they are critically important domains for elucidating the establishment of the canonical imprinted regions regulated by gDMRs. In addition, marsupials have other imprinted genes, such as *IGF2R* (Killian et al., 2000; Weidman et al., 2006; Suzuki et al., 2018) with a different gDMR from eutherians, and *PEG1/MEST* (Suzuki et al., 2005), with no apparent gDMR, and *INS,* which is adjacent to *IGF2* (Ager et al., 2007; Das et al., 2012). In total there are more than 10 marsupial-specific imprinted genes (Cao et al., 2023). Another paternally imprinted domain is the mouse-specific *Rasgrf1* imprinted domain with the gDMR comprised mostly of retrotransposons, LINEs, SINEs, LTRs and a copy of the solo-LTR RMER4B in the vicinity of a short tandem repeat sequence (Plass et al., 1996; Shibata et al., 1998; Watanabe et al., 2011).

Accumulating evidence on the molecular mechanisms underlying the establishment and maintenance of the DNA methylation marks on the gDMRs have further revealed the close relationship between genomic imprinting and invading DNA, such as retroviruses and LTR retrotransposons (Hanna and Kelsey, 2021; Kaneko-Ishino and Ishino, 2022). In addition to the insertion of DNA sequences corresponding to the gDMRs mentioned above, retroviral LTRs may serve as promoters of the oocyte-specific transcripts that are required for DNA methylation of the maternal gDMRs (Figure 9) (Chotalia et al., 2009; Veselovska et al., 2015; Brind’Amour et al., 2018; Bogutz et al., 2019; Smallwood et al., 2011; Hanna and Kelsey, 2021). In addition, the antiviral KRAB-ZFP system (Macfarlan et al., 2012; Rowe et al., 2013) protects the gDMRs from global DNA demethylation in the pre- and post-implantation stages (Edwards et al., 2019; Ondičova et al., 2020; Hanna and Kelsey, 2021). Thus, the eutharian genome used invading DNA as a genomic imprinting mechanism in a highly diverse variety of ways (Kaneko-Ishino and Ishino, 2022).

### 4.2 The relationship of *PEG10* and *PEG11/RTL1* to other SIRH/RTL genes

*PEG10* and *PEG11/RTL1* have nine homologs (RTL/SIRH genes) in eutherians (Brandt et al., 2005; Youngson et al., 2005; Ono et al., 2006) and 1 in marsupials (Ono et al., 2011). It is conceivable that all SIRH/RTL genes, including *PEG10* and *PEG11/RTL1,* originated from the same retrovirus with homology to the sushi-ichi retrotransposon. PEG10, PEG11/RTL1 and RTL3/SIRH9 have both GAG and POL-like domains, although the POL-like domain of RTL3/SIRH9 containing the DSG protease motif is shorter than PEG10 and PEG11/RTL1, while the other 8 RTL/SIRH genes encode only the GAG-like domain. It is difficult to determine whether all of the RTL/SIRH genes arose independently from individual retroviral integration events or whether some arose by cDNA retrotransposition from one or two common progenitors. *PEG10* may be the best ancestor candidate because it is the only gene conserved between marsupials and eutherians (Suzuki et al., 2007). *PEG11/RTL1* could be another candidate if it was indeed integrated in a common therian ancestor (Edwards, et al., 2008). It is possible that the GAG-like RTL/SIRH genes may have arisen as retrotransposed genes from the *PEG10*-ORF1 and/or *RTL3/SIRH9-*ORF1 cDNAs, or the partial cDNA of *PEG11/RTL1*.

Thus far, essential and/or important functions have been identified for 10 of the 11 RTL/SIRH genes (Kaneko-Ishino and Ishino, 2023). *RTL7/SIRH7* (formal gene name: *Leucine Zipper Downregulated in Cancer 1* (*LDOC1*)) is another essential placental gene that regulates trophoblast differentiation (Naruse et al., 2014). *RTL4/SIRH11* (aka *Zinc Finger CCHC Domain-Containint 16* (*ZCCHC16*) is implicated as a causative gene for autism spectrum disorders (ASD) (Lim et al., 2013) and *Sirh11/Zcchc16* KO mice exhibit increased impulsivity and diminished spatial memory, probably due to low levels of noradrenaline in the frontal cortex (Irie et al., 2015). *RTL8A, B, C/SIRH5, 6, 4* are triplet genes encoding almost identical proteins. Recently, it was reported that the RTL8 protein was found to accumulate together with the PEG10 protein in neuronal cells differentiated from iPS cells of Angelman syndrome patients (Pandya et al., 2021). In addition, *Rtl8a, b/Sirh5, 6* double KO mice exhibit late onset obesity and depression-like behavior, demonstrating they are important genes in the brain (Fujioka et al., 2023). *RTL6/SIRH3* (aka *LDOC1-like* (*LDOC1L*)) and the phylogenetically related *RTL5/SIRH8* (aka *Retrotransposon Gag Domain-Containing Protein 4* (*RGAG4*)) function as microglial genes in the innate immune system against bacteria and viruses by removing lipopolysaccharide and double stranded RNA from the brain, respectively (Irie et al., 2022). In addition, *RTL9/SIRH10* (aka *Retrotransposon Gag Domain-Containing Protein 1* (*RGAG1*) is also a microglial gene that is functionally active against fungi by reacting to zymosan, the cell wall of fungi (Ishino et al., 2023). Thus, at least three RLT/SIRH genes are involved in pathogen removal in the brain, including bacteria, viruses and fungi, suggesting that these genes must have substantially contributed to the evolution of the innate immune system in eutherians (Irie et al., 2022; Ishino et al., 2023).

These studies demonstrate that the exaptation (domestication) of these retrovirus-derived RTL/SIRH genes must have fundamentally contributed to the generation of certain eutherian-specific features in the placenta and brains. Where and how did these events happen in the course of development? Microglia originate from the yolk sac in early development and ultimately come to permanently reside in the brain. Therefore, it is of interest to notice that the placenta as well as the yolk sac, extraembryonic tissues in intrauterine development, seem to play a critical role as the original sites of exaptation of such retrovirus-derived genes. In the embryo, transcription of retroviruses and retrotransposons is completely repressed by DNA methylation, while the level of DNA methylation in these extraembryonic tissues is lower, thus allowing transcription. It is therefore likely that, via the selection of certain retrovirus-derived sequences, RTL/SIRH genes, including *PEG10* and *PEG11/RTL1,* and possibly some other eutherian-specific imprinted genes, such as *PEG3* and *PEG5/NNAT,* emerged as critically important players in the course of mammalian evolution (Kaneko-Ishino and Ishino, 2012; Kaneko-Ishino and Ishino, 2015; Irie et al., 2022; Ishino et al., 2023).

## 5 PEG10 and PEG11/RTL1 can form virus-like particles

It is known that the retroviral GAG protein is sufficient for the formation of virus particle proteins. Therefore, it is highly possible that some of the domesticated GAG-like proteins, such as PEG10 and PEG11/RTL1, can also self-assemble into virus-like particles (VLPs). One example of this is the Arc gene encoding GAG-like protein that may have originated from the Ty3/gypsy retrotransposon family (Ashley et al., 2018; Pastuzyn et al., 2018; Xie et al., 2018). The VLPs composed of the ARC protein bind to its own mRNA and may be able to transfer it to other cells. In the case of PEG10, Abed et el. first demonstrated that the mouse PEG10 GAG-like domain is able to form VLPs, and these can be encapsulated in a lipid bilayer and released outside of the cell (Abed et al., 2019). Segel et al. subsequently identified several proteins encoding the GAG-like domain, including both mouse and human PEG10 and mouse PEG11/RTL1, that were shown to be able to form VLPs by experiments using the overexpression of recombinant proteins (Segel et al., 2021). They also found that the VLP fractions purified after *Peg10* overexpression include appreciable amounts of *Peg10* mRNA, and the accumulation of the mRNA depends on the selectively of the binding of PEG10 to its own mRNA at the 5′UTR, the midpoint of its coding sequence and the beginning of the 3′ UTR.

Taking advantage of these unusual properties, they developed a novel specific RNA delivery system, called selective endogenous encapsidation for cellular delivery (SEND) (Segel et al., 2021). The SEND system is composed of 1) mouse PEG10 or human PEG10 as a GAG-like protein, 2) cargo mRNA consisting of both the 5′ and 3’ UTRs of mouse Peg10 or human PEG10 flanking a gene of interest and 3) envelope (ENV) or ENV-like protein as the fusogen required for fusing with target cells. The cells expressing all of these components produce PEG10 VLPs pseudotyped by the fusogen protein which packages the cargo mRNAs and mediates transfer of the RNA cargo into target cells.

Interestingly, the SYNA protein, which is encoded by domesticated *syncytin, a* gene evolved from retroviral *Env*, can function as a fusogen in the mouse SEND system. Given the facts that co-expression of *Peg10* and *Syna* is observed in some tissues and the treatment of primary mouse cortical neurons with mouse SYNA-pseudotyped PEG10 VLPs carrying native *Peg10* transcript as mRNA cargo induced both up- and downregulation of several genes, SYNA-pseudotyped PEG10 VLPs may be constantly produced in cells overexpressing the both genes and play a physiological role in normal cell activities.

## 6 Conclusion and outlook

Genomic imprinting is a critically important evolutionary feature of therian mammals. The placenta and highly developed brain are pivotal characteristics of the therian and eutherian mammals. It is highly likely that the retrovirus-derived *PEG10* and *PEG11/RTL1* were involved in these evolutionary modifications and powerfully contributed to the establishment of the current mammalian developmental system. However, many more unexpected functions will be discovered upon further investigation of *PEG10* and *PEG11/RTL1* as well as the RTL/SIRH genes, highlighting the central role of the exaptation of retrovirus-derived genes in mammalian development and evolution.
